# Plasma metabolome predicts trained immunity responses after antituberculosis BCG vaccination

**DOI:** 10.1371/journal.pbio.3001765

**Published:** 2022-09-12

**Authors:** Valerie A. C. M. Koeken, Cancan Qi, Vera P. Mourits, L. Charlotte J. de Bree, Simone J. C. F. M. Moorlag, Vidhisha Sonawane, Heidi Lemmers, Helga Dijkstra, Leo A. B. Joosten, Arjan van Laarhoven, Cheng-Jian Xu, Reinout van Crevel, Mihai G. Netea, Yang Li

**Affiliations:** 1 Department of Internal Medicine and Radboud Center for Infectious Diseases (RCI), Radboud Institute for Molecular Life Sciences (RIMLS), Radboud University Medical Center, Nijmegen, the Netherlands; 2 Department of Computational Biology for Individualised Infection Medicine, Centre for Individualised Infection Medicine (CiiM), a joint venture between the Helmholtz-Centre for Infection Research (HZI) and the Hannover Medical School (MHH), Hannover, Germany; 3 TWINCORE, a joint venture between the Helmholtz-Centre for Infection Research (HZI) and the Hannover Medical School (MHH), Hannover, Germany; 4 Department of Medical Genetics, Iuliu Haţieganu University of Medicine and Pharmacy, Cluj-Napoca, Romania; 5 Department for Immunology and Metabolism, Life and Medical Sciences Institute (LIMES), University of Bonn, Bonn, Germany; Duke University, UNITED STATES

## Abstract

The antituberculosis vaccine Bacillus Calmette–Guérin (BCG) induces nonspecific protection against heterologous infections, at least partly through induction of innate immune memory (trained immunity). The amplitude of the response to BCG is variable, but the factors that influence this response are poorly understood. Metabolites, either released by cells or absorbed from the gut, are known to influence immune responses, but whether they impact BCG responses is not known. We vaccinated 325 healthy individuals with BCG, and collected blood before, 2 weeks and 3 months after vaccination, to assess the influence of circulating metabolites on the immune responses induced by BCG. Circulating metabolite concentrations after BCG vaccination were found to have a more pronounced impact on trained immunity responses, such as the increase in IL-1β and TNF-α production upon *Staphylococcus aureus* stimulation, than on specific adaptive immune memory, assessed as IFN-γ production in response to *Mycobacterium tuberculosis*. Circulating metabolites at baseline were able to predict trained immunity responses at 3 months after vaccination and enrichment analysis based on the metabolites positively associated with trained immunity revealed enrichment of the tricarboxylic acid (TCA) cycle and glutamine metabolism, both of which were previously found to be important for trained immunity. Several new metabolic pathways that influence trained immunity were identified, among which taurine metabolism associated with BCG-induced trained immunity, a finding validated in functional experiments. In conclusion, circulating metabolites are important factors influencing BCG-induced trained immunity in humans. Modulation of metabolic pathways may be a novel strategy to improve vaccine and trained immunity responses.

## Abstract 2

The response to the BCG vaccine, which provides protection against tuberculosis as well as unrelated pathogens, is variable. This study shows that the baseline plasma metabolome is able to partially predict trained immunity upon BCG vaccination, identifying the metabolome as a potential source of this heterogeneity.

## Introduction

Certain microbial ligands and vaccinations can leave an immunological imprint on the innate immune system, leading to enhanced responsiveness later on. This de facto innate immune memory, which has been termed trained immunity, involves the functional reprogramming of innate immune cells such as monocytes and NK-cells [1]. Trained immunity can provide nonspecific protection against unrelated pathogens, as has been demonstrated for the antituberculosis vaccine Bacillus Calmette–Guérin (BCG) [2,3]. BCG vaccination results in enhanced ex vivo cytokine production by peripheral blood mononuclear cells (PBMCs) and NK-cells in response to unrelated pathogens such as *Staphylococcus aureus* and *Candida albicans* [4,5]. These trained immunity responses are regulated at epigenetic and transcriptional level [6,7], with metabolic changes being shown to be essential for the epigenetic modifications necessary for trained immunity [1]. BCG training up-regulates both glycolysis and oxidative phosphorylation in monocytes, and pharmacological inhibition of glycolysis results in abrogated trained immunity responses [8]. In vitro priming of monocytes with BCG or the fungal cell wall component β-glucan also activates glutaminolysis and accumulation of fumarate, which contributes to the induction of trained immunity by inhibition of the KDM5 histone demethylases [8,9].

In vivo responses to BCG vaccination are variable. Some individuals have a strong trained immunity and adaptive immune memory response after vaccination, while others respond poorly. This is evident in both experimental studies in which the epigenetic and immunological assessments have shown good responders and poor responders [10,11], as well as in the epidemiological studies that have shown a partial protection of BCG at populational level [12]. It is poorly understood which host and environmental factors account for the interindividual variability of immune responses to BCG vaccination. We hypothesized that interindividual differences in host metabolism and circulating metabolite concentrations influence this variability. In an effort to investigate this in vivo, we conducted a large cohort study in which we vaccinated more than 300 healthy individuals with BCG (300BCG study), followed by the assessment of the induction of trained immunity (heterologous induction of monocyte-derived cytokines in response to *S*. *aureus*) and adaptive immune memory (induction of T-cell responses by *Mycobacterium tuberculosis*) [13]. In this study, we examined if differential abundance of circulating metabolites affects induction of trained immunity and/or adaptive immune responses induced by BCG in vivo.

## Results

### Host factors associated with circulating metabolites

**Fig 1:**
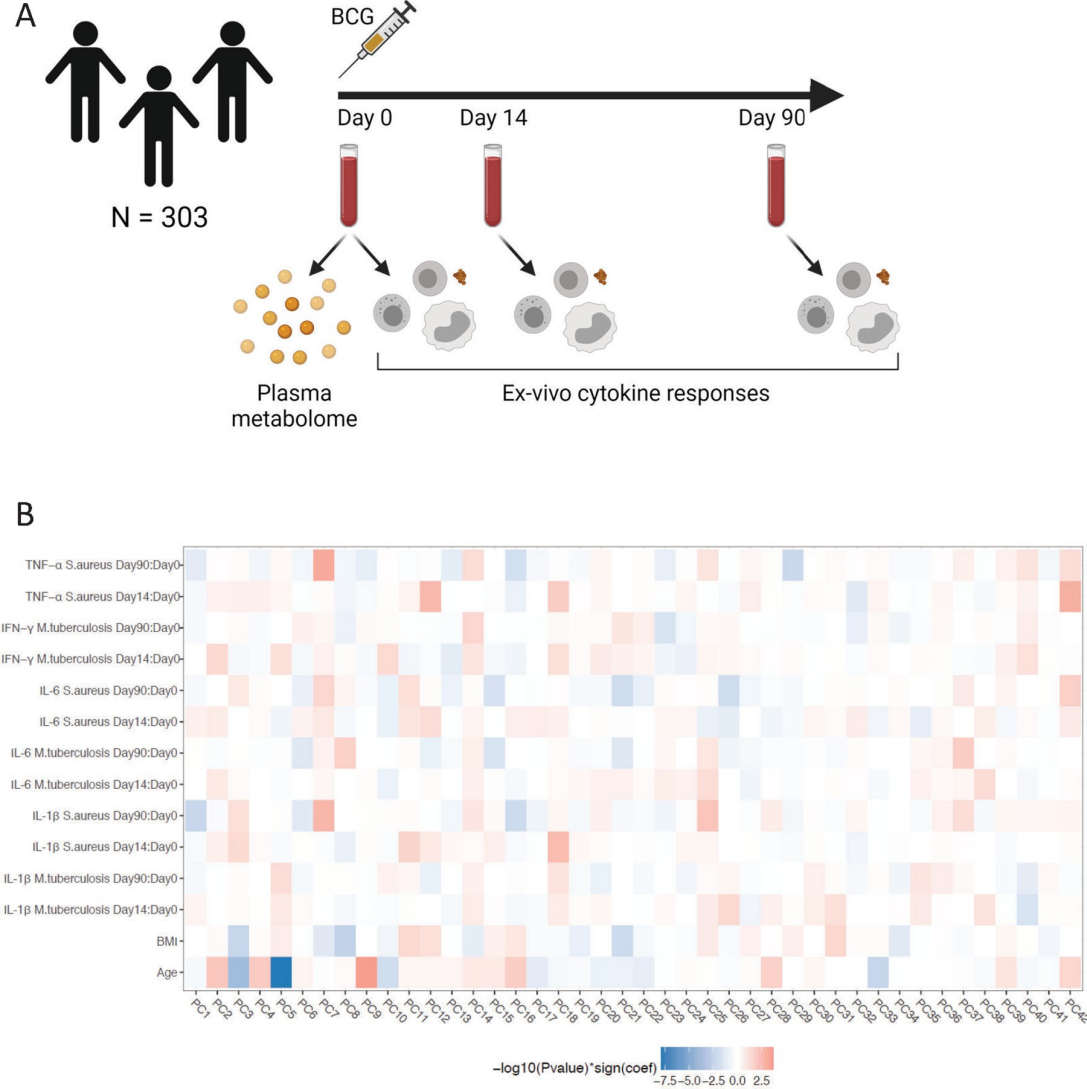
Study overview and correlation between circulating metabolome and ex vivo cytokine responses upon BCG vaccination. (**A**) Schematic overview of the study. Created with BioRender.com. (**B**) Heatmap of hierarchical clustering on Spearman correlation patterns between metabolites and PBMC-derived *S*. *aureus*-induced IL-1β, IL-6, and TNF-α responses and *M*. *tuberculosis*-induced IL-1β, IL-6, and IFN-γ responses. The ex vivo cytokine responses are analyzed as fold changes between baseline (v1, day 0) and 2 weeks (v2, day 14) and 3 months (v3, day 90) after BCG vaccination. The metabolites are represented as the top 42 PCs, capturing 75.3% of the variance. Cell colors indicate −log_10_(p-value) multiplied by the correlation coefficients from negative (blue) to positive (red). The metadata on study participants, metabolome, and cytokine data used to generate this figure are available at https://gitlab.com/xavier-lab-computation/public/bcg300. The loadings of the top 10 metabolites of the top 10 PC’s can be found in S1 Table. BCG, Bacillus Calmette–Guérin; PC, principal component; PBMC, peripheral blood mononuclear cell.

For a comprehensive measure of the circulating metabolome, flow-injection TOF-M was performed to profile a total of 1,607 metabolic features. After removing metabolites with an annotation score below 70, 1,373 features were left for further analysis. The metabolic measurements were performed in plasma samples from 325 healthy volunteers of Western European descent before BCG vaccination was administered (**Fig 1A**). Of the 325 participants, 18 were excluded because they received their vaccination in the evening, and we have learned that vaccination efficacy in the evening is suboptimal [14]. Four participants were excluded due to medication use or febrile illness, leaving 303 samples for further analysis. Of these 303 participants, 56% were female, the mean age was 23 years (range 18 to 71) and the mean body mass index (BMI) was 22.5 (±2.6 standard deviation).

We assessed the association between various host factors and the circulating metabolome. Sex and age both had a profound effect on circulating metabolites, with 35.3% of all metabolites significantly associated with sex and 38.2% of all metabolites associated with age (false discovery rate [FDR] <0.05, linear regression analysis). After correction for age and sex, only 0.95% of all metabolites were significantly associated with BMI and 0.22% of all metabolites were significantly associated with smoking. We also tested the association between metabolites and top 20 principal components (PCs) calculated from genotyping data, but no significant associations were identified at FDR <0.05. In female participants (*N* = 171), 17.0% of the metabolites were associated with oral contraceptive use. Vegetarian diet (reported by 8% of study subjects) was only associated with changes in concentrations of 0.36% of measured metabolites, among which methylhistidine as reported previously (FDR = 3.9 × 10^−3^, linear regression analysis, **S1 Fig**) [15].

### Baseline metabolites associated with innate and adaptive immune responses upon BCG vaccination

Next, we tested if circulating metabolites at baseline are associated with the immune response upon vaccination. To reduce data dimensionality, a principal component analysis (PCA) was performed on the circulating metabolome data (**S1 Table**). The first 42 PCs of the metabolic data were used for further analysis, capturing 75.3% of the total variance in the metabolome data (**S2 Fig**). Subsequently, we examined if these PCs representing the circulating metabolome predicted the innate and adaptive immune responses upon BCG vaccination. The immune response was assessed by ex vivo stimulation of PBMCs, and we assessed 2 immunological end points (**S3 Fig**):

- Trained immunity responses: comparison of cytokine production after heterologous stimulation with *S*. *aureus* of PBMCs isolated before and after BCG vaccination.
- Adaptive memory responses: comparison of the production of cytokines after *M*. *tuberculosis* ex vivo stimulation of PBMCs isolated before and after BCG vaccination.

To capture the interactions, Spearman correlations between the PCs representing the baseline metabolome and the ex vivo cytokine responses were calculated, and unsupervised hierarchical clustering analysis was performed on the correlation coefficients. Based on correlation patterns, several associations between metabolite modules and ex vivo cytokine responses upon BCG vaccination were found (**Fig 1B**). For example, PC7 was positively correlated with trained immunity responses, as measured by the increase in IL-1β, IL-6, and TNF-α production in response to *S*. *aureus* 3 months after BCG vaccination. The enriched metabolic pathways within the metabolite subset contributing to PC7 included arginine biosynthesis (*p* = 5.2 × 10^−3^, Fisher’s exact test), aminoacyl-tRNA biosynthesis (*p* = 7.3 × 10^−3^, Fisher’s exact test), and phenylalanine, tyrosine, and tryptophan biosynthesis (*p* = 4.1 × 10^−2^, Fisher’s exact test). Within the arginine biosynthesis metabolic pathway, metabolites including glutamine, fumarate, and aspartate contributed to PC7. In addition, PC5 and PC9 were highly correlated to age, again supporting the profound effect of age on the circulating metabolome.

**Fig 2:**
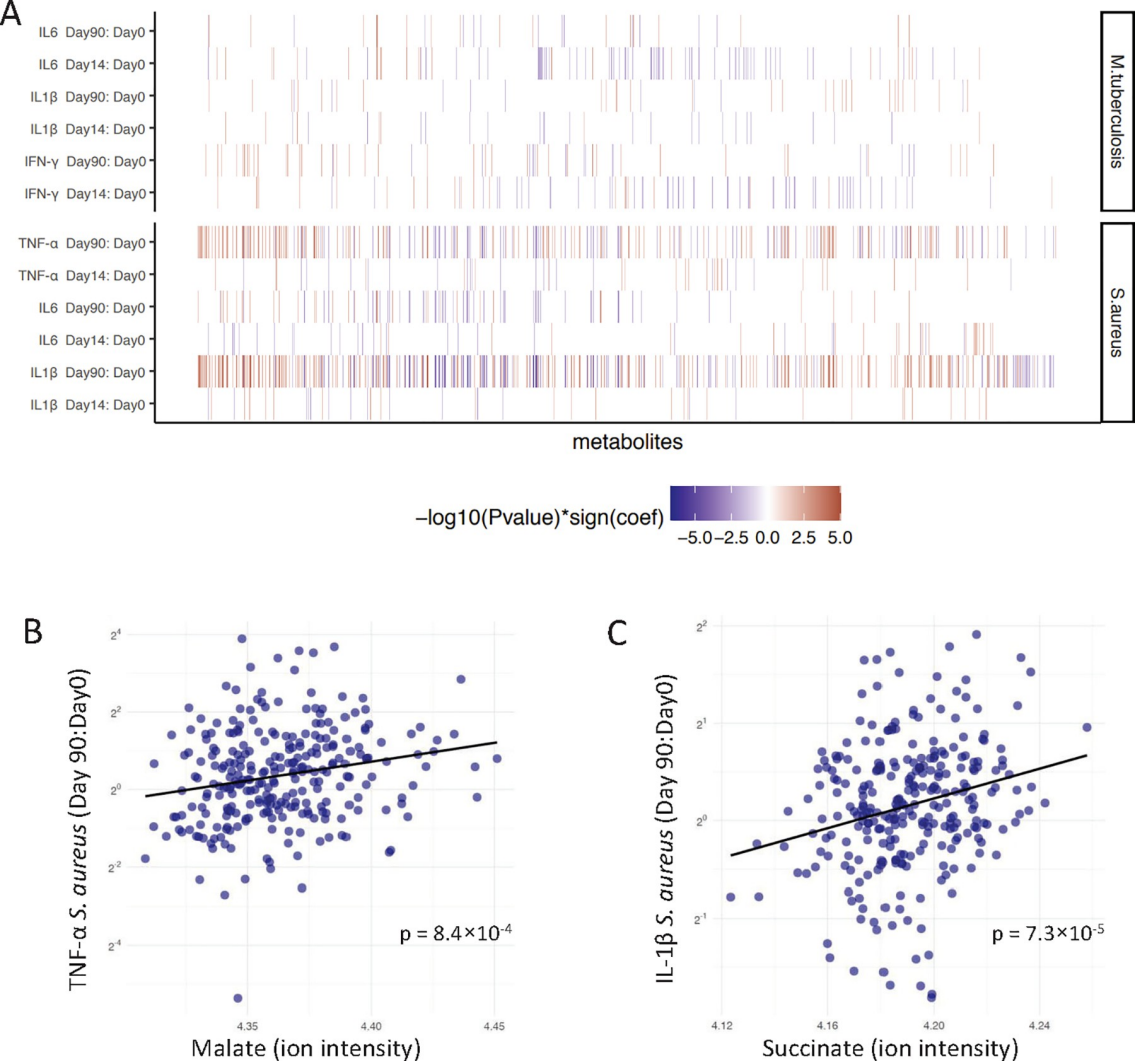
Association between baseline metabolites and ex vivo cytokine responses upon BCG vaccination. (**A**) Baseline metabolites were associated with fold changes of PBMC-derived *S*. *aureus*-induced IL-1β, IL-6, and TNF-α responses and *M*. *tuberculosis*-induced IL-1β, IL-6, and IFN-γ responses corrected for age and sex. Fold changes were calculated 2 weeks (v2, day 14) or 3 months (v3, day 90) after BCG vaccination compared to baseline (v1, day 0). Only metabolites with a significant association to one of the cytokine-stimulus pairs are depicted in this figure (*N* = 777, *p* < 0.05). The metabolites are ordered by hierarchical clustering. Cell colors indicate significant associations as −log_10_(p-value) multiplied by the correlation coefficients from negative (blue) to positive (red). The summary statistics of top 10 associated metabolites per stimulation can be found in S2 Table. (**B**) Linear regression between malate at baseline and fold changes of ex vivo PBMC-derived *S*. *aureus*-induced TNF-α responses 3 months after vaccination. (**C**) Linear regression between succinate at baseline and fold changes of ex vivo PBMC-derived *S*. *aureus*-induced IL-1β responses 3 months after vaccination. The metadata on study participants, metabolome, and cytokine data used to generate these figures are available at https://gitlab.com/xavier-lab-computation/public/bcg300. BCG, Bacillus Calmette–Guérin; PBMC, peripheral blood mononuclear cell.

To further test which metabolites contribute to ex vivo vaccine responses, we assessed the associations of all stimuli-cytokine combinations and individual metabolites corrected for age and sex (**S2 Table**). As shown in **Fig 2A**, baseline metabolite concentrations especially correlated with trained immunity responses, such as *S*. *aureus*-induced IL-1β and TNF-α production 3 months after BCG vaccination, rather than with specific adaptive immune memory, measured as *M*. *tuberculosis-*induced IFN-γ production (Fisher’s exact test, *p* = 5.0 × 10^−4^). Only 7% of the metabolites associated with the increase in IFN-γ in response to *M*. *tuberculosis* 3 months after BCG vaccination (*p* < 0.05), while 29% and 21% of the metabolites were associated with the increase in *S*. *aureus*-induced IL-1β and TNF-α, respectively. One of the key metabolites positively associated with IFN-γ responses induced by *M*. *tuberculosis* was 5-aminolevulinic acid, which is the first metabolite in the porphyrin synthesis pathway leading to the formation of heme [16]. Two important metabolites of the tricarboxylic acid (TCA) cycle, plasma malate and succinate, were strongly associated with increased production of TNF-α (*p* = 8.4 × 10^−4^, association with malate, linear regression analysis) and IL-1β (*p* = 7.3 × 10^−5^, association with succinate, linear regression analysis) after BCG vaccination, when PBMCs were stimulated with *S*. *aureus* (**Fig 2B** and **2C**). Nonlinear regression, used to test the robustness of this linear relationship, showed similar results as the linear regression model (**S4 Fig** and **S3 Table**).

### Baseline metabolites predict trained immunity responses upon BCG vaccination

**Fig 3:**
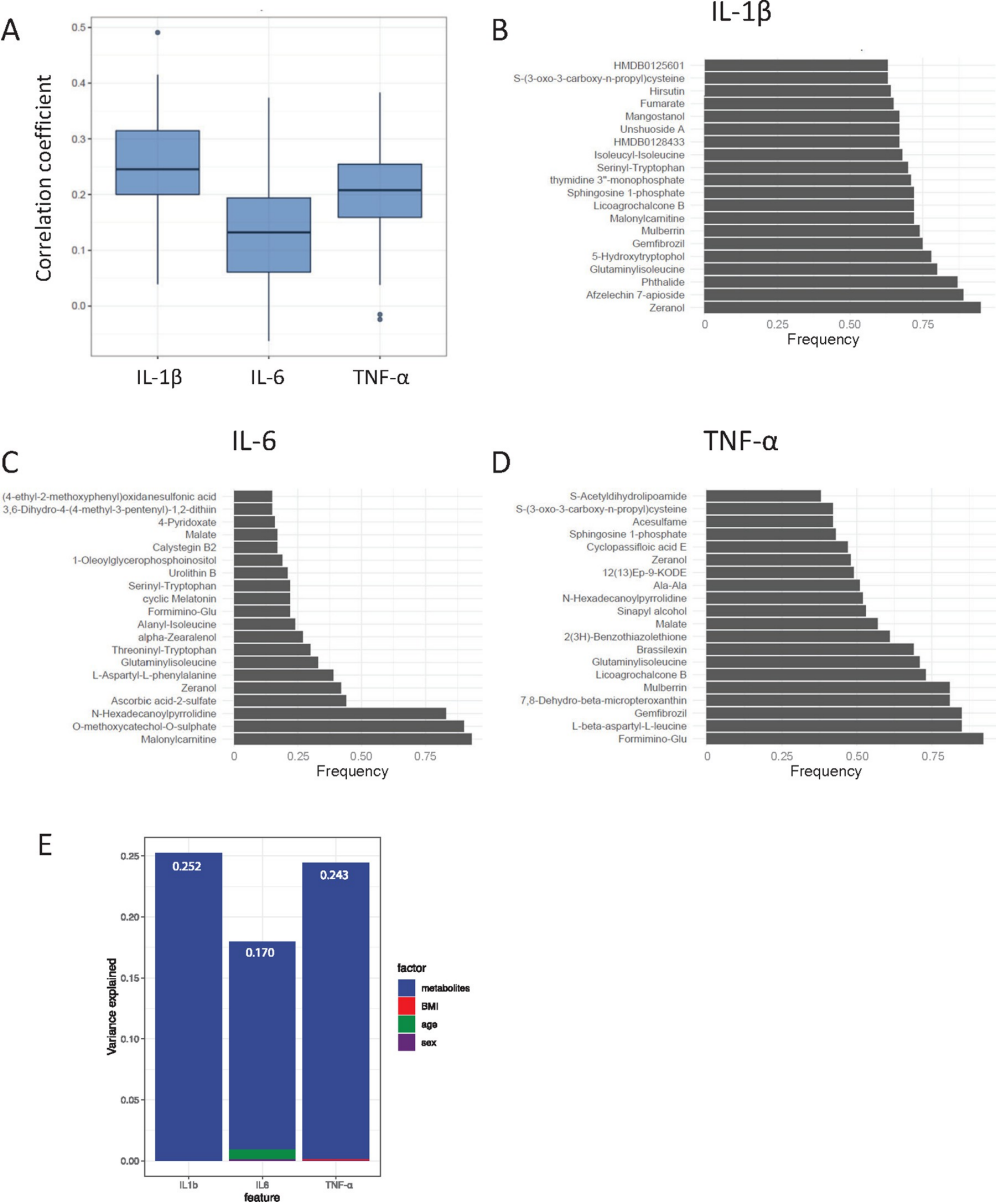
Baseline metabolites predict trained immunity responses 3 months after BCG vaccination. (**A**) The dataset was randomly split into a training set (70% of the observations) and a test set (30%). Then, a 10-fold cross-validation was performed for feature selection, which was repeated 5 times, to generate a prediction model for IL-1β, IL-6, and TNF-α fold changes in response to *S*. *aureus* 3 months after BCG vaccination. Prediction accuracy was evaluated by calculation of a Pearson correlation between the measured cytokine fold changes and the predictions of the test sets. This procedure was repeated 100 times, and the correlation coefficients are visualized as boxplots. (**B–D**) The top 20 metabolites occurring in the prediction models for IL-1β (**B**), IL-6 (**C**), and TNF-α (**D**) fold changes in response to *S*. *aureus* 3 months after BCG vaccination are visualized. In total, 100 models were constructed for each cytokine fold change. (**E**) Variance explained by metabolites (top 10 most often occurring metabolites from the prediction analysis), age, sex, and BMI on trained immunity responses. Values indicate the variance explained by metabolites. The metadata on study participants, metabolome, and cytokine data used to generate these figures are available at https://gitlab.com/xavier-lab-computation/public/bcg300. BCG, Bacillus Calmette–Guérin; BMI, body mass index.

Next, we assessed the predictive ability of baseline metabolites for the trained immunity responses. In this analysis, we aimed to predict the fold changes of *S*. *aureus*-induced cytokines 3 months after vaccination, as a large proportion of baseline metabolites were associated with these responses. First, the dataset was randomly split into a training set (70% of the data) and a test set (30%), after which 10-fold cross-validation was performed to select features, which was repeated 5 times, using Elastic Net [17]. Then, the accuracy of each model was tested by calculating a Pearson correlation between the predicted values based on the model and the measured values in the test dataset (30% of the observations), as a measure of the similarity between the predicted and measured cytokine fold changes. This whole procedure was repeated 100 times, generating 100 models. The correlation coefficients, as a measure of the prediction performance of these models, are visualized in **Fig 3A**. The median correlations between predicted and measured cytokine fold changes were 0.25 for IL-1β, 0.13 for IL-6, and 0.21 for TNF-α. Thus, the predictive power of baseline metabolites for trained immunity responses was relatively low, although statistically significant. The predictive power and stability of the model can probably be increased by including multi-omics data from different layers, e.g., epigenetics [18]. Then, we analyzed which metabolites occurred most frequently in the prediction models for IL-1β (**Fig 3B**), IL-6 (**Fig 3C**), and TNF-α (**Fig 3D**) in response to *S*. *aureus* 3 months after vaccination. Interestingly, metabolites of the TCA cycle again appeared in our results, as fumarate was 65 out of 100 times selected in the model to predict higher IL-1β production, and malate was selected 57 times to predict higher TNF-α production. As previously described, fumarate itself is able to induce trained immunity in an in vitro setting (**S5 Fig**), whereas malate is not [9]. Other metabolites that occurred in high frequency were malonyl carnitine, which is an acyl carnitine; formiminoglutamic acid, also known as formimino-glu, which is an intermediate in the catabolism of histidine; and 5-hydroxytryptophol, which is a serotonin metabolite. We observed that metabolites explained a larger percentage of the variation in trained immunity responses than age, sex, and BMI (**Fig 3E**). The top 10 metabolites from each rank list from the prediction results explained 25.2% variance for IL-1β, 17.0% for IL-6, and 24.3% for TNF-α fold changes in response to *S*. *aureus*.

### Metabolite co-accumulation networks associated with trained immunity responses

**Fig 4:**
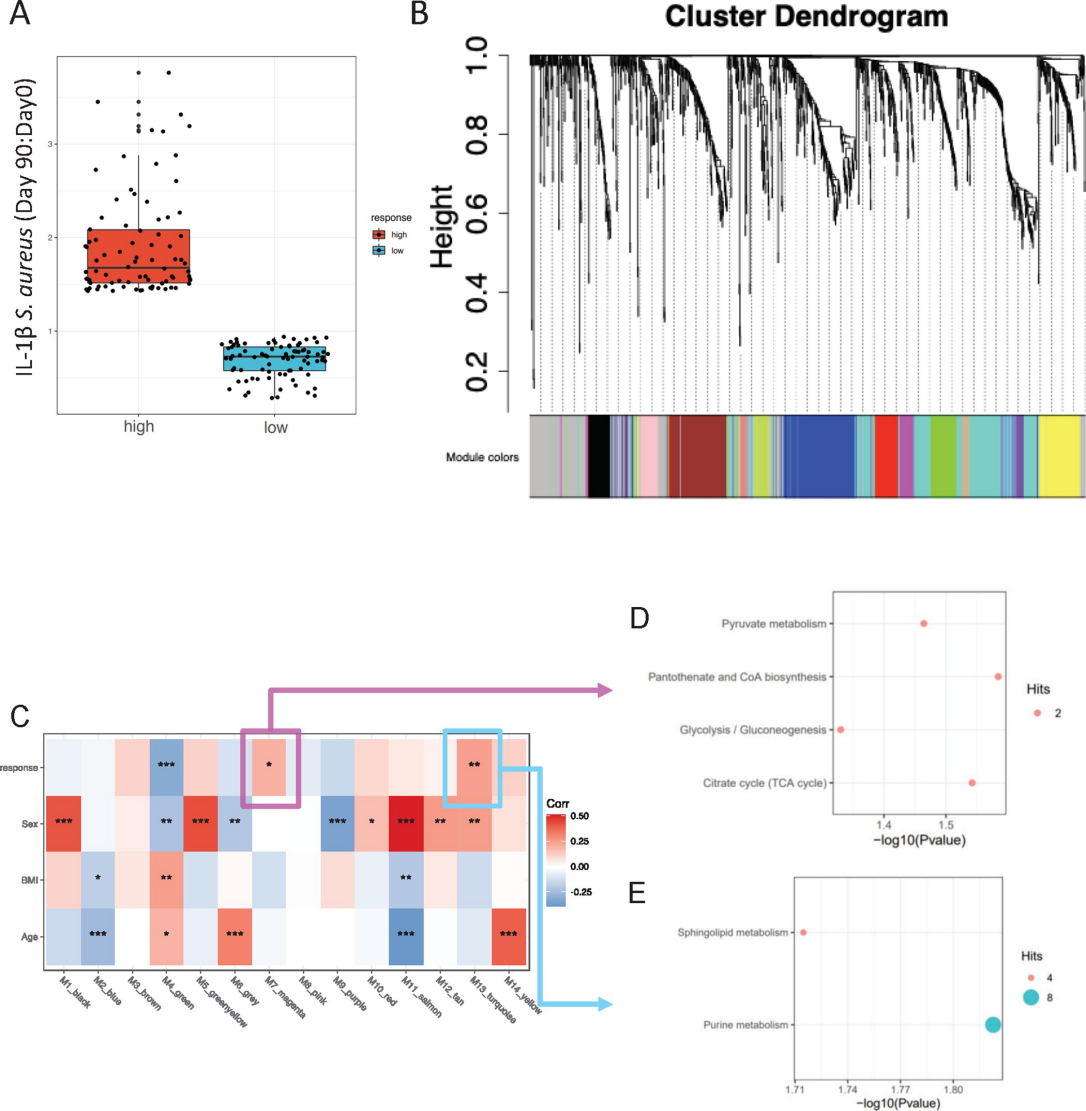
Co-accumulated metabolites modules associated with trained immunity response level. (**A**) Distribution of high and low responders based on the fold change in the ex vivo production of IL-1β in response to *S*. *aureus* 3 months after vaccination compared to baseline. (**B**) WGCNA was performed on metabolites from high and low responders of trained immunity. The metabolites included in each module identified by WGCNA can be found in S4 Table. (**C**) The identified 14 modules were correlated to high/low responder classification, age, sex, and BMI, respectively. Stars indicate significant correlation with * *p* < 0.05, ** *p* < 0.01, *** *p* < 0.001. (**D** and **E**) Dot plots showing results of pathways analysis of metabolites from module magenta (**D**) and turquoise (**E**). The summary statistics can be found in S6 Table. No significant pathways from metabolites of the green module were identified. The metadata on study participants, metabolome, and cytokine data used to generate these figures are available at https://gitlab.com/xavier-lab-computation/public/bcg300. BMI, body mass index; WGCNA, weighted correlation network analysis.

Considering the interindividual variation in trained immunity responses induced by BCG, both high and low responders can be identified based on the increase in ex vivo cytokine responses. To identify metabolic networks underlying the responder phenotype, we assessed the metabolites co-accumulation networks in high and low responders in terms of their trained immunity response (as defined by the fold change in IL-1β after *S*. *aureus* stimulation, **Fig 4A**) and identified 14 modules of highly correlated metabolites (**Fig 4B** and **S4 Table**). Subsequently, we correlated the 14 modules to trained immunity responses (high/low), age, sex, and BMI. Three modules (green, magenta, and turquoise) were associated with trained immunity response (**Fig 4C**); in a sensitivity analysis adjusting for age, sex, and BMI, the same 3 modules were associated with trained immunity (**S5 Table**). Metabolites from module magenta were enriched for the pantothenate and CoA biosynthesis (*p* = 0.026) and TCA cycle pathways (*p* = 0.029, **Fig 4D** and **S6 Table**), again linking TCA metabolites to BCG-induced trained immunity responses. In addition, metabolites from module turquoise were enriched in purine and sphingolipid metabolism (**Fig 4E** and **S6 Table**). We did not observe any pathways enriched based on the metabolites of the green module.

### Metabolic pathways and taurine metabolism associated with BCG-induced trained immunity

**Fig 5:**
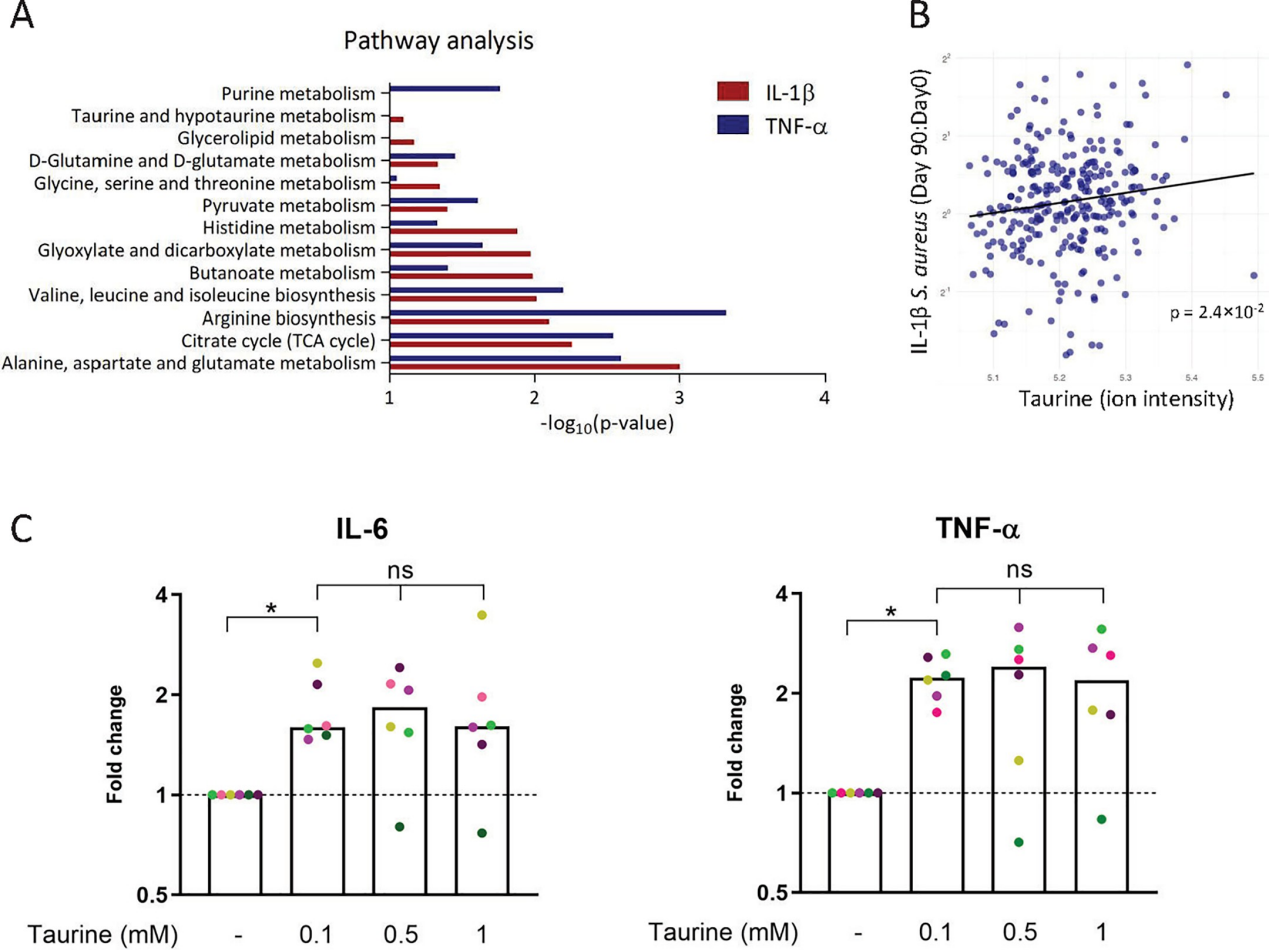
Pathway analysis of metabolites associated with trained immunity and the effect of taurine. (**A**) Pathway analysis of metabolites that are significantly (*p* < 0.05) associated with the fold change in IL-1β and TNF-α in response to *S*. *aureus* 3 months after BCG vaccination. Pathway analyses were performed using MetaboAnalyst (KEGG version October 2019) [19]. (**B**) Linear regression between taurine at baseline and fold changes of ex vivo PBMC-derived *S*. *aureus*-induced IL-1β responses 3 months after vaccination. The metadata on study participants, metabolome, and cytokine data used to generate these figures are available at https://gitlab.com/xavier-lab-computation/public/bcg300. (**C**) Human primary monocytes were incubated for 24 hours with taurine (0.1, 0.5, or 1 mM), after which the medium was refreshed, and the cells were allowed to rest for 5 days, after which they were stimulated with *E*. *coli* LPS (10 ng/mL) for 24 hours. Then, the levels of IL-6 and TNF-α were measured, and a fold change was calculated relative to the medium control. The median values are presented, and each donor is represented in a different color (*N* = 6, Wilcoxon matched-pairs signed rank test, ns = not significant, * *p* < 0.05). The cytokine values used to generate this figure can be found in **S2 Data**. BCG, Bacillus Calmette–Guérin; PBMC, peripheral blood mononuclear cell.

To identify the metabolic pathways involved, we included all metabolites associated with the ex vivo cytokine responses upon BCG vaccination (linear regression analysis with age and sex as covariates, *p* < 0.05) in subsequent pathway analyses. The pathway analyses were run independently for each cytokine-stimulus combination per time point and separately for negative and positive associations. The pathways enriched for metabolites positively associated with IL-1β and TNF-α in response to *S*. *aureus* 3 months after BCG vaccination are visualized in **Fig 5A**.

For the first time, we show that many of the pathways that were implicated in trained immunity from previous in vitro trained immunity studies [9,10], such as TCA cycle, glutaminolysis, and pyruvate metabolism, are also associated with BCG-induced trained immunity in vivo. One of the pathways that has not been previously implicated in trained immunity was taurine metabolism. Taurine was positively associated with induction of trained immunity, as assessed by fold-increase in IL-1β (**Fig 5B**) and TNF-α (**S6 Fig**) in response to *S*. *aureus* 3 months after BCG vaccination (linear regression analysis, all mentioned associations *p* < 0.05). To investigate a functional link between taurine and trained immunity, we performed an in vitro trained immunity experiment in which we primed human monocytes with taurine, followed by restimulation of these cells with LPS after 5 days of rest [19]. Indeed, preincubation of cells with taurine led to induction of trained immunity, as shown by increased IL-6 and TNF-α production upon restimulation with LPS, which are the commonly used read-outs to assess trained immunity in vitro (**Fig 5C**). Interestingly, however, taurine did not amplify the induction of trained immunity induced by BCG or β-glucan (**S7 Fig**). None of the tested conditions affected cell viability after priming (**S8 Fig**).

Considering the significant impact of meat and fish consumption on metabolic profiles, including taurine [20], we next examined if the consumption of meat or fish was associated with altered ex vivo cytokine responses upon BCG vaccination. However, reported consumption of meat and fish (reported as consumption days per week) showed no relation with ex vivo cytokine responses upon BCG vaccination, possibly since day-to-day variation in food intake was not captured in our questionnaire.

## Discussion

BCG vaccination induces heterologous protection against infectious diseases other than tuberculosis, at least in part through induction of trained immunity [1,10]. Induction of trained immunity leads to enhanced responsiveness of innate immune cells and is dependent on epigenetic and metabolic reprogramming. BCG effectiveness to induce trained immunity is widely heterogeneous between individuals [10,11], but the source of this variation is not known. We hypothesized that circulating metabolites are an important factor influencing both innate and adaptive memory responses, and that they represent an important source of variation in vaccine responses between individuals. Indeed, in a large cohort of vaccinated healthy volunteers, we observed that the circulating metabolomic signature predicts BCG-induced immune responses, especially trained immunity responses.

A large number of circulating metabolites were associated with trained immunity responses, and our in vivo study validates several findings from previous in vitro studies. For example, previous in vitro studies have shown that glutamine metabolism is increased upon BCG training and that inhibition of glutaminolysis leads to decreased trained immunity responses [8]. At the same time, metabolites that accumulate upon glutaminolysis in trained cells, such as fumarate, can induce trained immunity through inhibition of KDM5 demethylase, and increase in H3K4me3 at the promotors of immune genes [9]. These in vitro data are validated by our study, in which we also observed that metabolites involved in glutamine and glutamate metabolism (such as succinate and malate) are associated with BCG-induced trained immunity. In addition, succinate was previously shown to be involved in innate immune signaling and related to enhanced IL-1β responses in macrophages [21]. These data argue that variation in the concentrations of TCA metabolites in the circulation represents an important factor in the response to BCG vaccination.

Interestingly, the association between circulating metabolites and BCG-induced immune responses was much stronger for heterologous *S*. *aureus*-induced cytokine responses at 3 months rather than 2 weeks, especially for IL-1β and TNF-α. Although we observe an increase in cytokine responses at both 2 weeks as well as 3 months after vaccination, the early time point is probably more reflective of acute infection, as live BCG is still detectable 2 weeks after vaccination [22], while the 3-month time point truly reflects innate immune memory.

We wanted to select for further validation and discussion novel pathways that based on biological arguments would likely be involved in trained immunity, rather than already known metabolites previously shown to modulate it. We therefore selected taurine metabolism for further exploration because little is known about its role in BCG-induced trained immunity, for its known anti-inflammatory effects, and since it can be found in high concentrations in phagocytes. Taurine, or 2-aminoethanesulfonic acid, is a metabolite widely present in animal tissue that is very important for cardiovascular function but also for the development of muscles, retina, and nervous tissue. Taurine can be produced endogenously, but it is also obtained from diet: both meat and fish are rich in taurine, while a vegan diet provides almost no taurine. Interestingly, taurine has been reported to have anti-inflammatory effects by inhibition of cytokine production [23,24] and is present in high concentrations in phagocytes, especially in neutrophils, where it has both anti-inflammatory and antioxidant functions [25,26]. In our study, taurine concentrations were associated with trained immunity induced by BCG vaccination, and additional in vitro experiments revealed that taurine is able to induce trained immunity itself, without any microbial ligands present. This is the first study implicating a potential role for taurine in trained immunity, and its mechanism of action needs to be investigated in future studies. Regarding the impact of taurine, a limitation of the current study is that we could validate the induction of trained immunity by taurine itself, but in the in vitro experimental model, taurine did not further increase the trained immunity induced by BCG. We hypothesize that in the in vitro model, the induction of trained immunity by BCG is already very effective, reaching a maximum that cannot be further increased by taurine. The mechanism through which taurine induces trained immunity does not seem to be as acting as a pathogen-associated molecular pattern (PAMP), as taurine itself (without LPS restimulation) did not induce cytokine production. This is similar to the effects of fumarate, which does not act as a PAMP, but as an inhibitor of epigenetic enzymes (in that case KDM5 histone demethylase), as shown by Arts and colleagues [9]. Unfortunately, very little is known about the impact of taurine on epigenetic processes. We have indeed attempted to assess it by using general inhibitors of histone methylation such as methyltioadenosine (MTA); however, MTA exerted similar effects on cytokine induction by LPS either with or without taurine (not shown), arguing that taurine exerts its effects through a different mechanism. The molecular mechanism through which taurine exerts its effect needs to be comprehensively investigated in future studies.

A surprising observation from our study is that the circulating metabolome seems to have a more pronounced effect on trained immunity responses compared to the adaptive immune responses induced by BCG vaccination. This may seem unexpected, as cellular metabolism is important for T-cell function [27]. While cellular metabolism is important for both innate and adaptive immune responses, it seems that the baseline abundance of metabolites more strongly affects variation in innate rather than adaptive immune responses induced by BCG. These results are in line with a recent study that observed a stronger effect of baseline metabolites on interindividual variation of monocyte-derived cytokines (TNF-α, IL-1β, IL-6) compared to T cell-derived cytokines (IL-17, IL-22, IFN-γ) [28]. Various metabolites influence the activity of epigenetic modifying enzymes, for example, acetyl-CoA, which acts as an acetyl group donor for histone acetyltransferases [29]. Since epigenetic remodeling is the molecular basis for trained immunity, this might explain why the abundance of certain metabolites has a pronounced effect on BCG-induced trained immunity. The observation that metabolites show less association with IFN-γ production in response to *M*. *tuberculosis* is also interesting for tuberculosis vaccine development, as this suggests that the metabolome might not be a major factor influencing the variable protection provided by BCG against tuberculosis. However, our lack of understanding of correlates of protection in the context of vaccination against TB makes it difficult to evaluate these results in the context of tuberculosis vaccination [30].

Our study is limited by the fact that the observed link between metabolome and trained immunity relies on associative analyses (with the exception of taurine), and it remains to be investigated if there exists a causal relationship. In addition, the observations from this study should be validated in an independent cohort of BCG-vaccinated individuals. Assessment of intracellular cytokine staining in addition to the measurement of secreted cytokines would further enrich the dataset of the study, but we were unfortunately unable to perform these experiments due to logistical limitations. Considering that BCG is mainly administered in neonates in tuberculosis endemic countries, it remains to be investigated if these results can be translated to different populations. Additionally, the pathway enrichment analyses performed can assist interpretation and connection of metabolites that were associated with BCG-induced immune responses. However, it is important to realize that these are cellular metabolism pathways, and that some metabolites important for cellular metabolism are not measurable in plasma, and that not all circulating metabolites are part of cellular metabolism pathways. Furthermore, although we were able to measure and analyze a comprehensive set of 1,373 metabolites, which enables us to perform unbiased analyses, the flow injection platform is unable to discriminate and identify isomers. Our study identified potentially important metabolic processes for BCG-induced trained immunity, which should be validated by targeted metabolomics.

In conclusion, the findings from this study confirm the role of circulating metabolites on BCG-induced trained immunity in vivo. In addition, we identified taurine metabolism as a potential pathway linking metabolism and epigenetic alterations in trained immunity. These data suggest that metabolite supplementation or metabolic modulation could be used in the future to improve vaccine responses and prevent infectious diseases.

## Methods

### Study design

To study the innate and adaptive immune responses upon BCG vaccination, healthy volunteers of Western European ancestry were included in the 300BCG cohort between April 2017 and June 2018, as previously described [13]. The study was approved by the Arnhem-Nijmegen Medical Ethical Committee (NL58553.091.16) and performed in accordance with the declaration of Helsinki. After written informed consent was obtained, a standard dose of 0.1 mL BCG (BCG-Bulgaria, InterVax) was administered intradermally in the left upper arm. EDTA blood was collected before and 2 weeks and 3 months after BCG vaccination. Volunteers were not eligible for participation in case of systemic medication use other than oral contraceptives or acetaminophen, use of antibiotics 3 months before inclusion, previous BCG vaccination, any febrile illness 4 weeks before participation, any vaccination 3 months before participation, or a medical history of immunodeficiency. Participants were also not eligible if they recalled a history of tuberculosis or had lived in a tuberculosis-endemic setting. No tuberculosis skin test or IFN-γ release assay was performed to test for latent tuberculosis infection, but the tuberculosis incidence in the indigenous Dutch population is extremely low (1/100,000) [31]. After BCG administration, the study participants received an extensive online questionnaire about lifestyle, diet, and disease history. This questionnaire included questions about meat and fish intake, which was categorized into daily, weekly, monthly, less than once a month, or never, and can be found in **S7 Table**.

### Peripheral blood mononuclear (PBMC) cell isolation and stimulation

PBMCs were isolated from whole blood using Ficoll-Paque (GE healthcare) density gradient separation. PBMCs were washed twice with phosphate-buffered saline (PBS) and resuspended in Dutch modified RPMI 1640 medium (Roswell Park Memorial Institute, Invitrogen), supplemented with 50 μg/mL gentamicin, 2 mM Glutamax (GIBCO), and 1 mM pyruvate (GIBCO). In a total volume of 200 μL/well, 5 × 10^5^ PBMCs were cultured in round bottom 96-well plates (Greiner) and stimulated with RPMI (medium control), heat-killed *M*. *tuberculosis* H37Rv (5 μg/mL), or heat-killed *S*. *aureus* (10^6^ CFU/mL) and incubated at 37°C with 5% CO_2_. Supernatants were collected after 24 hours and 7 days and stored at −20°C until further analysis. IL-1β, IL-6, and TNF-α (ELISA, R&D Systems) concentrations were measured after 24 hours, and IFN-γ (Luminex, Thermo Fisher) was measured after 7 days, according to the manufacturer’s protocols.

### Metabolomics measurement

Plasma metabolite levels were measured before BCG vaccination was administered. The metabolic features were measured and annotated by the General Metabolics (Zurich, Switzerland) using flow injection time-of-flight mass (flow-injection TOF-M) spectrometry, as previously described [32]. Non-targeted metabolites were annotated according to human metabolites database (HMDB). All metabolomic measurements were performed in duplicate, and the average value was calculated for each sample per feature.

### In vitro trained immunity assay

The trained immunity assay was performed as previously described [19,33]. Briefly, buffy coats from healthy donors were obtained after written informed consent (Sanquin Blood Bank, Nijmegen, the Netherlands). PBMCs were isolated using Ficoll-Paque, after which Percoll isolation was performed to enrich the monocyte fraction. The cells were allowed to adhere for 1 hour, after which the non-adherent cells were washed away with PBS. Then, the adherent monocytes were preincubated with various concentrations of taurine (0.1, 0.5, or 1 mM; mean concentration of taurine in blood in adults is 93 μM [34]), methyl-fumarate (0.1 mM) or left untreated, after which they were incubated for 24 hours in medium (negative control), BCG (SSI, 5 μg/mL), or β-glucan (1 μg/mL). Cell viability was assessed using CytoTox 96 non-radioactive cytotoxicity assay (Promega), which measures the cytosolic enzyme lactate dehydrogenase (LDH) that is released upon cell lysis. After 24 hours, the cells were washed with PBS and incubated in medium for 5 days. Subsequently, the cells were restimulated with medium alone or ultra-pure *Escherichia coli* LPS (10 ng/mL), after which the concentrations of IL-6 and TNF-α were determined in the supernatants (ELISA, R&D Systems).

### Data analyses

All computational analyses were performed using R version 3.3.3. The Shapiro–Wilk test was applied to check for a normal distribution of cytokine and metabolite levels. Preprocessing analysis includes log transformation of the cytokine and metabolite data and filtering of metabolites with an annotation score <70. We studied the effect of age, sex, BMI, and oral contraceptive usage on metabolite levels using a linear regression model, and a FDR was calculated to adjust for multiple testing. To reduce data dimensionality, we performed a PCA on baseline metabolites using centered and scaled data. The PCs capturing at least 75% of the variance were included in the analysis to represent the metabolome data. Spearman correlation analysis was performed on metabolite PCs and ex vivo cytokine fold changes, and unsupervised hierarchical clustering was performed, which was visualized in a heatmap (**Fig 1B**). Ex vivo cytokine fold changes were associated with metabolites using a linear regression model with age and sex as covariates (**Fig 2**).

Next, we assessed the predictive value of baseline metabolites to predict the trained immunity responses (ex vivo cytokine fold changes in response to *S*. *aureus*). Prediction of the cytokine fold changes was performed by training an Elastic Net model, which is a regularization and variable selection method especially useful when the number of predictors is larger than the number of observations [17]. First, the dataset was randomly split into a training set (70% of the data) and a test set (30%), after which a 10-fold cross-validation was performed to select features, which was repeated 5 times, based on the training set. Prediction accuracy was evaluated by calculation of a Pearson correlation between the measured cytokine fold changes and the predictions of the Elastic Net model on the test sets. Then, this procedure was repeated 100 times, and the results were summarized (**Fig 3A–3D**).

Subsequently, we estimated the variance explained by metabolites, age, sex, and BMI on cytokine fold changes. To assess the contribution of each type of data upon the others, the full model (included age, sex, BMI, and top 10 metabolites) was fit first. Subsequently, several reduced models were fit in which 1 data level was missing. The difference of R^2^ between the reduced model and the full model was taken as a measure of the variance explained by that level when accounting for the effects of the other levels (**Fig 3E**) [18].

To understand the role of metabolite networks in high and low responders in terms of their trained immunity response, we first defined the high and low responders by ranking subjects based on their IL-1β fold change in response to *S*. *aureus*. The top 30% subjects were defined as high responders and the bottom 30% as low responders (**Fig 4A**). Metabolite data from both high and low responders were merged together, and we performed weighted correlation network analysis (WGCNA) [35] to identify modules of highly correlated metabolites (**Fig 4B**). Then, we correlated the summary profile (eigengene) for each module with the clinical traits (high/low responders, age, sex, BMI; **Fig 4C**). If we identified any modules that correlated with high/low responders, we then extracted the metabolites from this module and performed pathway analysis by MetaboAnalyst 5.0 (https://www.metaboanalyst.ca/; KEGG version October 2019) [36], and metabolite pathways with *p* < 0.05 were reported (**Fig 4D** and **4E**).

Finally, all metabolites with a *p* < 0.05 from the linear regression models were included in further pathway analysis using MetaboAnalyst (**Fig 5A**) [36]. Meat and fish intake was reported by study participants as daily, weekly, monthly, or never, and the ex vivo cytokine fold changes between these groups were compared using Kruskal–Wallis tests.

## Supporting information

S1 Fig Methylhistidine levels at baseline compared between vegetarians (*N* = 24) and non-vegetarians (*N* = 279, FDR = 3.9 × 10^−3^). The metadata on study participants and metabolome data used to generate this figure are available at https://gitlab.com/xavier-lab-computation/public/bcg300.
(TIF)

S2 Fig Scree plot of the explained variance per PC capturing the metabolomics data. The figure visualizes the first 42 PCs, which capture 75.3% of the total variance in the metabolome data. The metabolome data used to generate this figure are available at https://gitlab.com/xavier-lab-computation/public/bcg300.
(TIF)

S3 Fig Log2 fold change of ex vivo PBMC-derived cytokine responses. PBMCs were stimulated with *S*. *aureus* or *M*. *tuberculosis* and IL-6, IL-1β, and TNF-α were measured in the supernatant after 24 hours and IFN-γ after 7 days. The fold change calculates the response at day 14 or day 90 (after vaccination) compared to day 0 (before vaccination). The cytokine data used to generate this figure are available at https://gitlab.com/xavier-lab-computation/public/bcg300.
(TIF)

S4 Fig Association between malate at baseline and fold changes of ex vivo PBMC-derived *S*. *aureus*-induced TNF-α responses (left) and between succinate at baseline and fold changes of ex vivo PBMC-derived *S*. *aureus*-induced IL-1β responses (right) 3 months after vaccination. The blue line and shadow indicate the linear regression model and the red line and shadow indicate the spline regression model. The cytokine and metabolome data used to generate this figure are available at https://gitlab.com/xavier-lab-computation/public/bcg300.
(TIF)

S5 Fig Human primary monocytes were incubated for 24 hours with fumarate (0.1 mM) or BCG (1 μg/mL), after which the medium was refreshed, and the cells were allowed to rest for 5 days, after which they were stimulated with *E*. *coli* LPS (10 ng/mL) for 24 hours. Then, the levels of IL-6 and TNF-α were measured, and a fold change was calculated relative to the medium control. The median values are presented (*N* = 6 [IL-6] and *N* = 3 [TNF-α], Wilcoxon matched-pairs signed rank test, * *p* < 0.05). The cytokine values used to generate this figure can be found in **S1 Data**.
(TIF)

S6 Fig Linear regression between taurine at baseline and fold changes of ex vivo PBMC-derived *S*. *aureus*-induced TNF-α responses 3 months after vaccination. The cytokine and metabolome data used to generate this figure are available at https://gitlab.com/xavier-lab-computation/public/bcg300.
(TIF)

S7 Fig Human primary monocytes were preincubated in the presence of taurine (0.1, 0.5, or 1 mM) for 1 hour, after which they were incubated for 24 hours with culture medium, BCG (5 μg/mL), or β-glucan (1 μg/mL). After 24 hours, the medium was refreshed, and the cells were allowed to rest for 5 days, after which they were stimulated with *E*. *coli* LPS (10 ng/mL) for 24 hours. Then, the levels of IL-6 and TNF-α were measured, and a fold change was calculated relative to the medium control. The median values are presented, and each donor is represented in a different color (*N* = 6, Wilcoxon matched-pairs signed rank test, ns = not significant, * *p* < 0.05). The cytokine values used to generate this figure can be found in **S3 Data**.
(TIF)

S8 Fig Cytotoxicity assay that measures the cytosolic enzyme LDH that is released upon cell lysis to test the cell viability after priming. Human primary monocytes were preincubated in the presence of taurine (0.1, 0.5, or 1 mM) for 1 hour, after which they were incubated for 24 hours with culture medium, BCG (5 μg/mL), or β-glucan (1 μg/mL). After 24 hours, LDH was measured in the supernatant (*N* = 6 per condition). The y-axis represents the degree of cytotoxicity in percentage relative to the positive and negative control. The cytotoxicity values used to generate this figure can be found in **S4 Data**.
(TIF)

S1 Table Loadings of top 10 metabolites with high contribution to each PC (PC1-10). (XLSX)

S2 Table Summary statistics of top 10 associated metabolites per stimulation. (XLSX)

S3 Table Anova test of linear regression and spline model. (XLSX)

S4 Table Metabolites included in each module identified by WGCNA. (XLSX)

S5 Table Sensitivity analysis of association between high responders/low responders and modules adjusted for age, sex, and BMI. (XLSX)

S6 Table Enrichment analysis for modules related to trained immunity. (XLSX)

S7 Table Digital survey questions. (XLSX)

S1 Data Raw data underlying the summary data displayed in S5 Fig. (XLSX)

S2 Data Raw data underlying the summary data displayed in Fig 5C. (XLSX)

S3 Data Raw data underlying the summary data displayed in S7 Fig. (XLSX)

S4 Data Raw data underlying the summary data displayed in S8 Fig. (XLSX)

## Abbreviations

BCG: Bacillus Calmette–Guérin
BMI: body mass index
FDR: false discovery rate
HMDB: human metabolites database
LDH: lactate dehydrogenase
MTA: methyltioadenosine
PAMP: pathogen-associated molecular pattern
PBS: phosphate-buffered saline
PC: principal component
PCA: principal component analysis
PBMC: peripheral blood mononuclear cell
TCA: tricarboxylic acid
WGCNA: weighted correlation network analysis
