# Supplementary figures and images for: Plasma metabolome predicts trained immunity responses after antituberculosis BCG vaccination
Source: PLoS Biol. 2022 Sep 12;20(9):e3001765. doi: 10.1371/journal.pbio.3001765 (PMC9499240; doi:10.1371/journal.pbio.3001765)

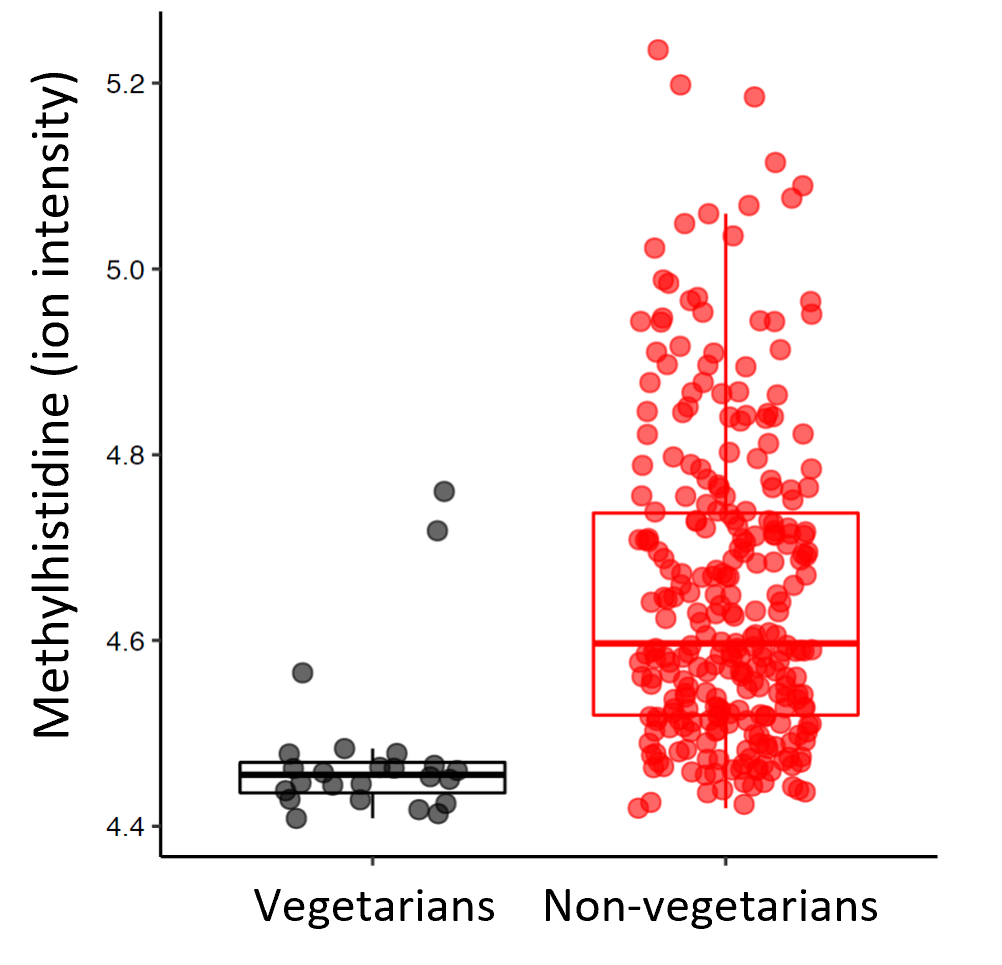

Supplement: S1 Fig — The metadata on study participants and metabolome data used to generate this figure are available at https://gitlab.com/xavier-lab-computation/public/bcg300. (TIF) [file pbio.3001765.s001.tif]

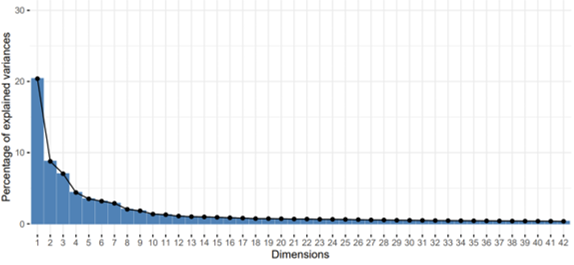

Supplement: S2 Fig — The figure visualizes the first 42 PCs, which capture 75.3% of the total variance in the metabolome data. The metabolome data used to generate this figure are available at https://gitlab.com/xavier-lab-computation/public/bcg300. (TIF) [file pbio.3001765.s002.tif]

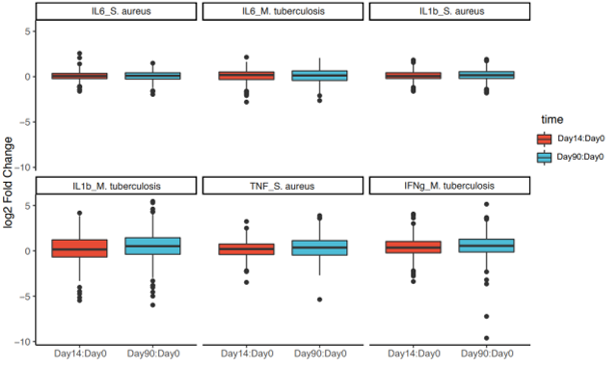

Supplement: S3 Fig — PBMCs were stimulated with S. aureus or M. tuberculosis and IL-6, IL-1β, and TNF-α were measured in the supernatant after 24 hours and IFN-γ after 7 days. The fold change calculates the response at day 14 or day 90 (after vaccination) compared to day 0 (before vaccination). The cytokine data used to generate this figure are available at https://gitlab.com/xavier-lab-computation/public/bcg300. (TIF) [file pbio.3001765.s003.tif]

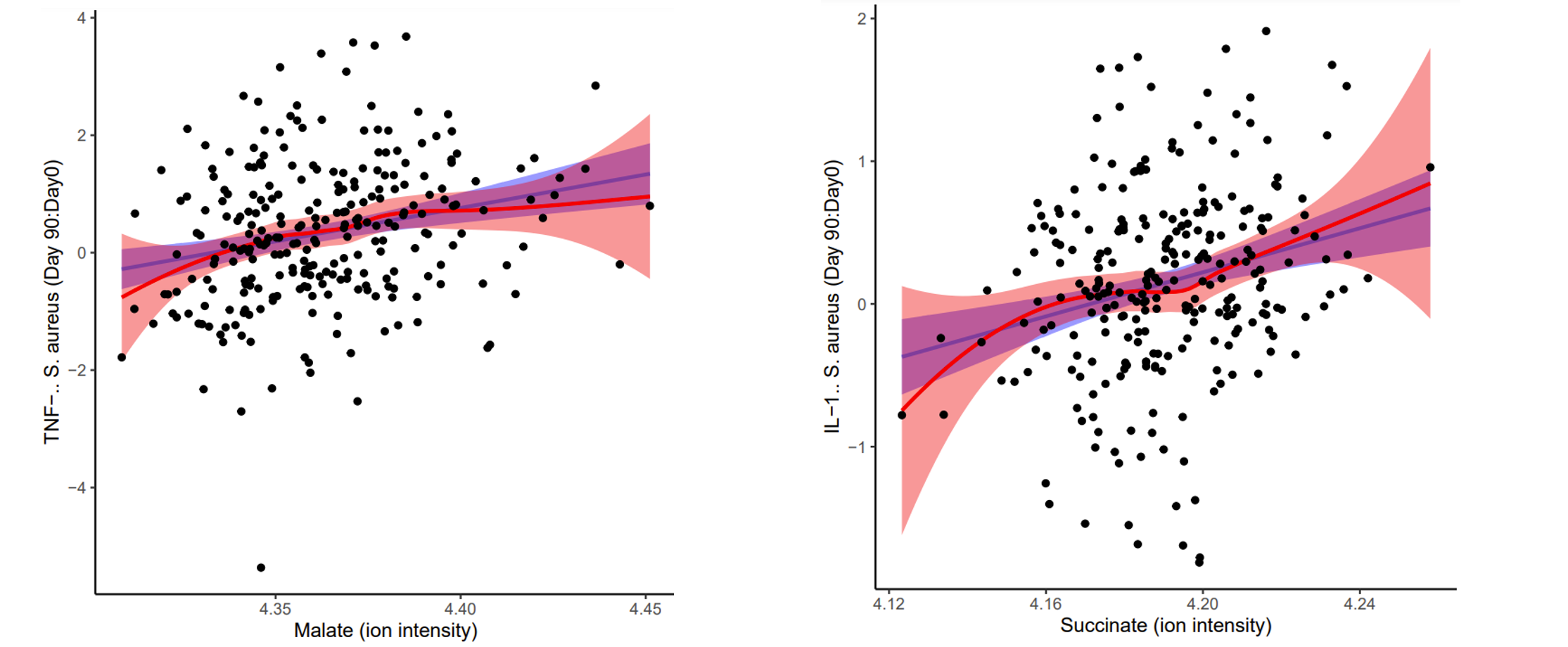

Supplement: S4 Fig — The blue line and shadow indicate the linear regression model and the red line and shadow indicate the spline regression model. The cytokine and metabolome data used to generate this figure are available at https://gitlab.com/xavier-lab-computation/public/bcg300. (TIF) [file pbio.3001765.s004.tif]

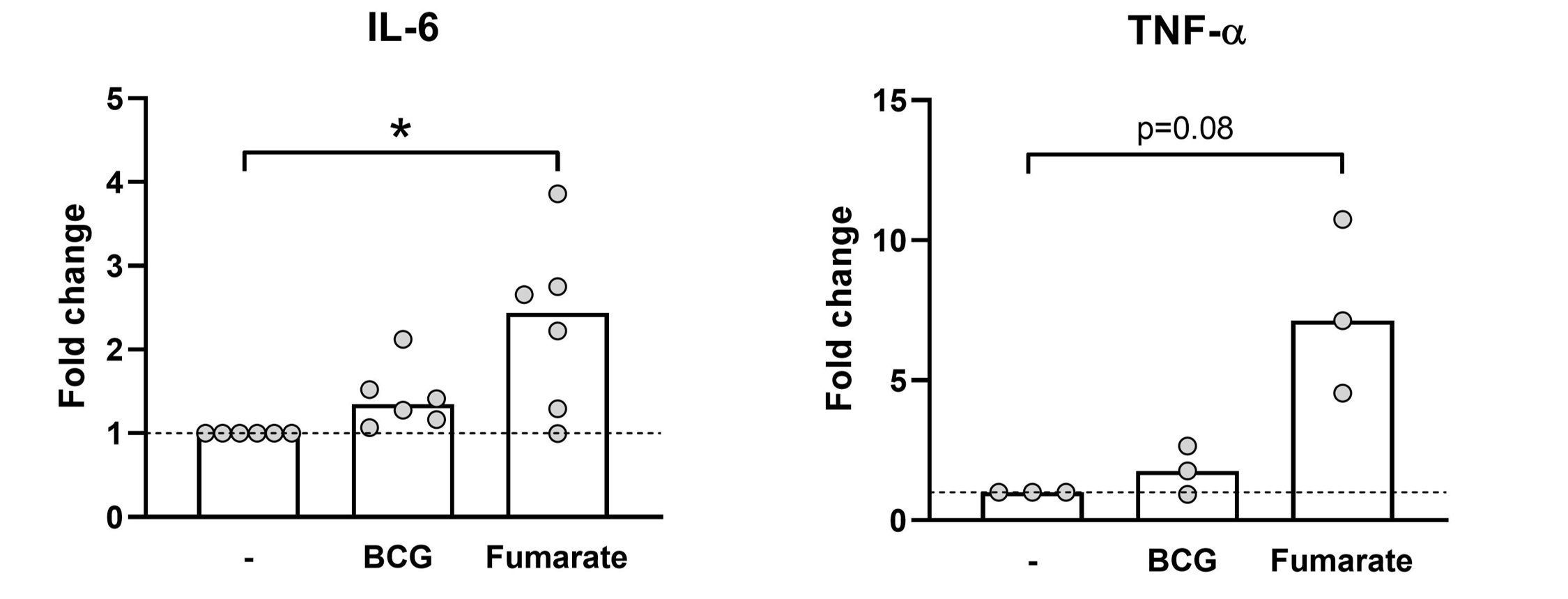

Supplement: S5 Fig — Then, the levels of IL-6 and TNF-α were measured, and a fold change was calculated relative to the medium control. The median values are presented (N = 6 [IL-6] and N = 3 [TNF-α], Wilcoxon matched-pairs signed rank test, * p < 0.05). The cytokine values used to generate this figure can be found in S1 Data. (TIF) [file pbio.3001765.s005.tif]

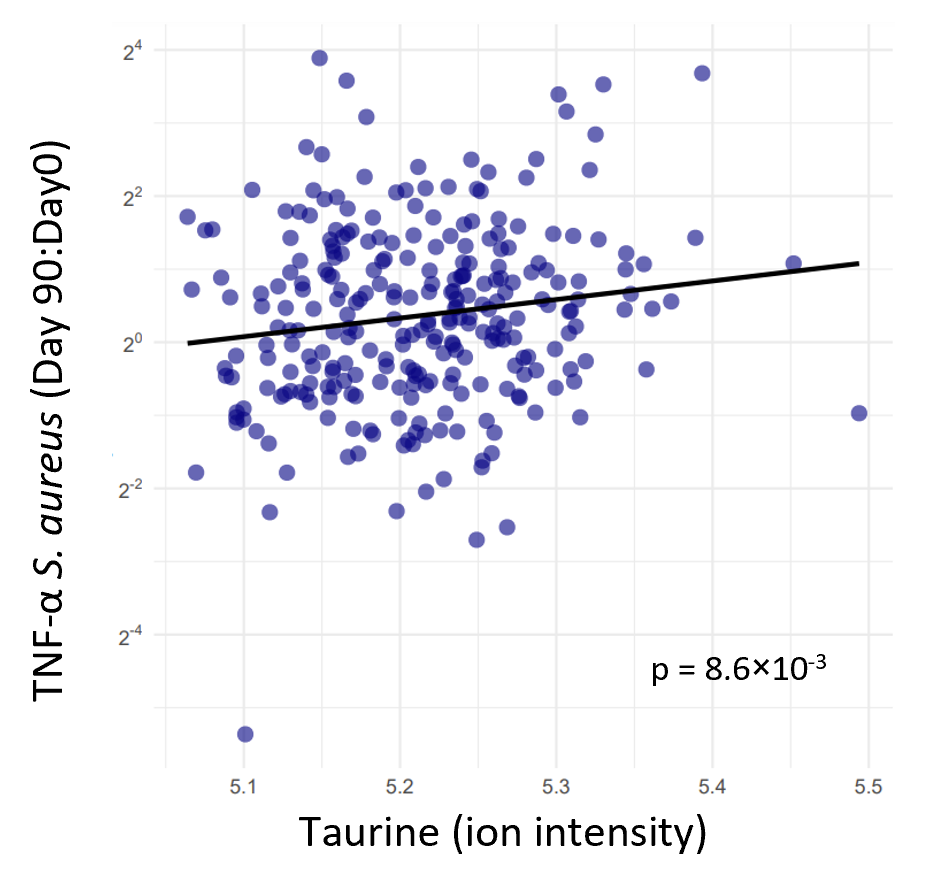

Supplement: S6 Fig — The cytokine and metabolome data used to generate this figure are available at https://gitlab.com/xavier-lab-computation/public/bcg300. (TIF) [file pbio.3001765.s006.tif]

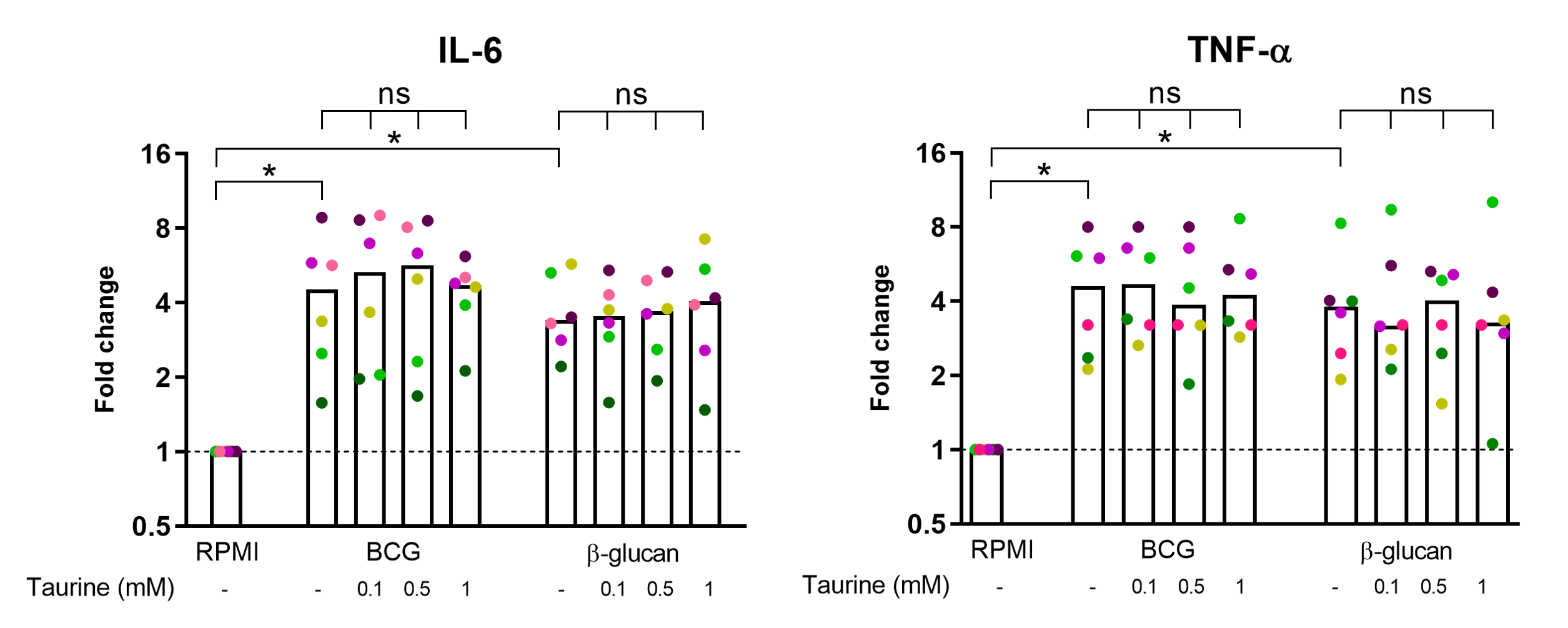

Supplement: S7 Fig — After 24 hours, the medium was refreshed, and the cells were allowed to rest for 5 days, after which they were stimulated with E. coli LPS (10 ng/mL) for 24 hours. Then, the levels of IL-6 and TNF-α were measured, and a fold change was calculated relative to the medium control. The median values are presented, and each donor is represented in a different color (N = 6, Wilcoxon matched-pairs signed rank test, ns = not significant, * p < 0.05). The cytokine values used to generate this figure can be found in S3 Data. (TIF) [file pbio.3001765.s007.tif]

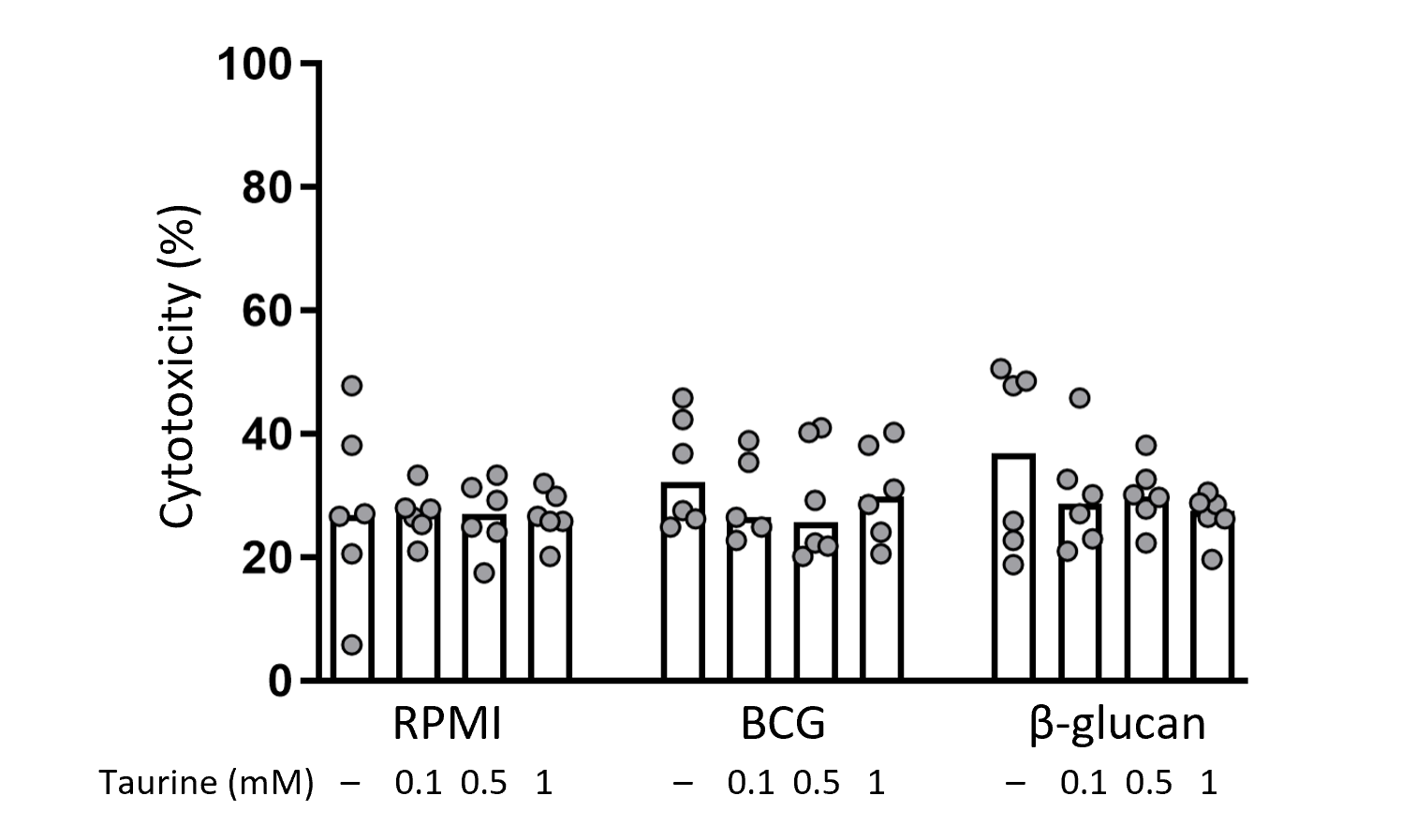

Supplement: S8 Fig — Human primary monocytes were preincubated in the presence of taurine (0.1, 0.5, or 1 mM) for 1 hour, after which they were incubated for 24 hours with culture medium, BCG (5 μg/mL), or β-glucan (1 μg/mL). After 24 hours, LDH was measured in the supernatant (N = 6 per condition). The y-axis represents the degree of cytotoxicity in percentage relative to the positive and negative control. The cytotoxicity values used to generate this figure can be found in S4 Data. (TIF) [file pbio.3001765.s008.tif]
